# Salivary Melatonin Changes in Oncological Patients: A Systematic Review

**DOI:** 10.3390/metabo12050439

**Published:** 2022-05-13

**Authors:** Kacper Nijakowski, Michał Surdacki, Małgorzata Sobieszczańska

**Affiliations:** 1 Department of Conservative Dentistry and Endodontics, Poznan University of Medical Sciences, 60-812 Poznan, Poland; 2 Student Research Group of Geriatrics and Gerontology, Department of Clinical Geriatrics, Wroclaw Medical University, 50-369 Wroclaw, Poland; michal.surdacki@student.umw.edu.pl; 3 Department of Clinical Geriatrics, Wroclaw Medical University, 50-369 Wroclaw, Poland; malgorzata.sobieszczanska@umw.edu.pl

**Keywords:** melatonin, saliva, oncological diagnostics, sleep quality, circadian rhythm, brain tumours, prostate cancer, oral squamous cell carcinoma, lung cancer, lymphoma

## Abstract

Melatonin is known as a regulator of circadian sleep and waking rhythm. This hormone secreted by the pineal gland also has protective, oncostatic, and antioxidant properties. This systematic review was designed to answer the question “Is there a relationship between salivary melatonin changes and oncological diseases?”. Following the inclusion and exclusion criteria, ten studies were included, according to PRISMA statement guidelines. In all included studies, the diagnostic material was unstimulated whole saliva, in which the melatonin changes were determined by different laboratory methods. Most studies concerned changes in melatonin levels in patients with brain tumours due to a direct effect on the circadian rhythm centres. Other studies focused on disorders of melatonin secretion and its inclusion as a diagnostic marker in patients with prostate cancer and oral squamous cell carcinoma. The association between melatonin changes and sleep quality and chronotype in patients with newly diagnosed lung cancer and lymphoma survivors was also investigated. In conclusion, our systematic review may suggest trends for melatonin secretion alterations in oncological patients. However, due to the significant heterogeneity of the included reports, it is not possible to clearly determine a link between changes in salivary melatonin levels and the oncological diagnosis.

## 1. Introduction

The ubiquitous artificial light pollution interferes with the natural daily cycle of humans and inhibits melatonin secretion [1]. Blue light waves play a special role in long-term exposure. The main source of blue light is sunlight, but lighting technology in the form of light-emitting diodes (LEDs) evolved considerably over the past 20 years. Diodes have advantages over previous conventional incandescent lamps due to their longer lifetime, lower operating costs, and lower energy consumption; on the other hand, they emit more blue light. Blue light, mainly with a wavelength of 460–480 nm, inhibits melatonin biosynthesis proportional to light intensity and exposure length [2].

The physiological production of melatonin decreases with age, and the maximum plasma value reaches between 3 and 4 h at night. The light/dark cycle is the main system that regulates its release [3]. Short wavelengths corresponding to blue light have a much stronger damping effect on melatonin levels than longer waves, e.g., orange waves emitted by the fire. After exposure to light over several consecutive nights, a melatonin secretion is not only stopped during the night but postponed to the morning hours [4].

Melatonin is a stronger antioxidant than vitamin E and has a strong anti-cancer effect. This hormone is also an immunomodulator, and synthesis inhibition causes a suppressed humoral and cellular response [5,6,7,8].

Among other things, melatonin increases the expression of the two circadian genes *Per1* and *Per2*, which inhibit the proliferation of cancer cells by stimulating their apoptosis. The *Per* genes are considered to be suppressor genes of breast cancer cells that interact with estrogen receptor compounds [9]. An elevated risk of breast cancer has been shown to depend on shiftwork. For every ten years of shiftwork, a 16% increase in cancer risk was shown, which is associated with longer exposure to blue light at night [10]. Melatonin can be used as an adjunct to the treatment of breast cancer and has been shown to be a strong inhibitor of aromatase activity by affecting cyclooxygenase [11]. Administration of melatonin during chemotherapy reduced the incidence of thrombocytopenia, neurotoxicity, cardiotoxicity, mucositis, and asthenia [12].

In the case of prostate cancer, melatonin can stimulate the immune system by increasing the production of interleukin-2 and interleukin-4 by Th lymphocytes; as with breast cancer, it can protect DNA from oxidative damage by balancing free radicals [13]. Melatonin’s nocturnal hormone signal has been shown to modulate the initiation, progression, and growth of prostate tumours by, among other things, inhibiting the uptake of linoleic acid used by prostate cancer cells and blocking the Warburg effect, which prevents the cells from growing. However, these effects disappear when melatonin production is suppressed during light exposure at night [14].

Determination of salivary melatonin is associated with the growing diagnostic potential of this biological material and the advantages of its collection. The choice of saliva instead of serum or urine is supported by the invasiveness of sampling and the relatively high simplicity of the examination. In addition, salivary levels reflect the secretory profile of the nocturnal melatonin rhythm [15]. The assessment of the saliva metabolome changes allows the detection and differentiation of tumour lesions, e.g., oral squamous cell carcinoma [16].

Our systematic review was designed in order to answer the question “Is there a relationship between salivary melatonin changes and oncological diseases?”, formulated according to PICO (“Population”, “Intervention”, “Comparison”, and “Outcome”).

## 2. Results

**Figure 1:**
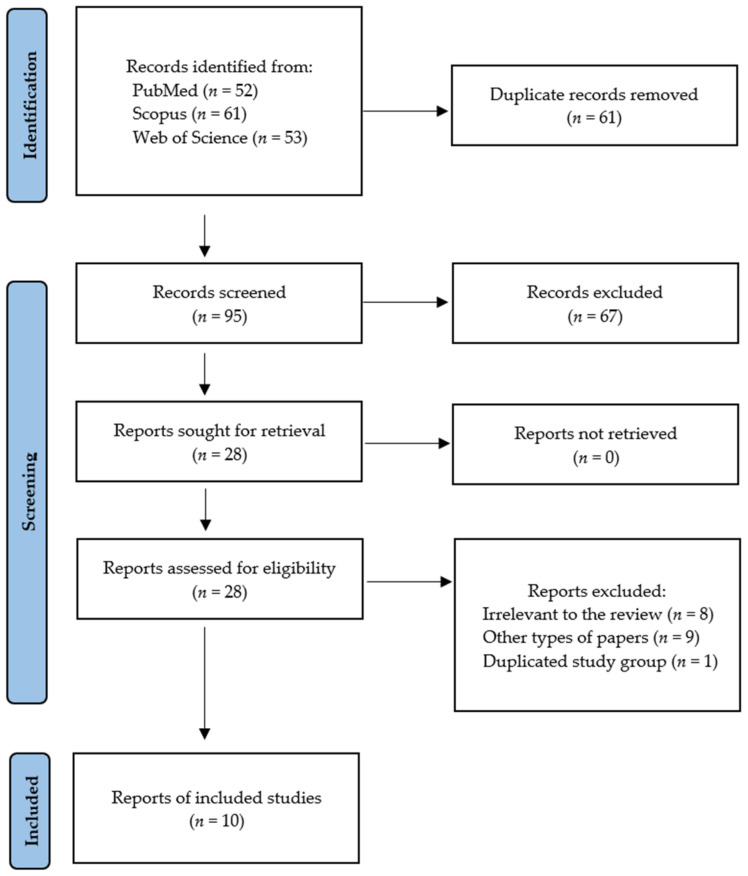
PRISMA flow diagram presenting search strategy.

Following the search criteria, our systematic review included ten studies, demonstrating data collected in seven different countries from a total of 371 participants with diagnosed oncological diseases (including 129 females and 242 males), and 221 controls (including 54 females and 167 males). Figure 1 shows the detailed selection strategy of the articles. The inclusion and exclusion criteria are presented in Table 1.

From each eligible study included in the present systematic review, we collected data about its general characteristics, such as year of publication and setting, involved participants, oncological diagnosis, inclusion and exclusion criteria, and assessed sleep parameters (Table 2). Table 3 presents the detailed characteristics considering types of saliva, methods of collection, centrifugation, storing and laboratory analysis for salivary melatonin, as well as other determined markers. All of the studies took into consideration unstimulated whole saliva samples. Saliva centrifugation methods were often not reported. Moreover, methods of sample storing were rather heterogeneous; freezing conditions were approximately evenly distributed for −20 and −80 °C. Additionally, the most important findings about salivary melatonin from included studies are displayed in Table 4.

## 3. Discussion

The use of saliva to assess melatonin levels has only been increasing in recent years. Non-invasive saliva collection allows the determination of dim light melatonin onset, for which this biological material is well restricted. The concentration of salivary melatonin seems to reflect the level of free circulating melatonin as opposed to serum, in which it occurs in a bound form with relatively low affinity to albumin and higher affinity to alpha-1-acid glycoprotein [27]. The determination of melatonin levels may also be influenced by the circadian rhythm, the salivary flow rate, and the measurement method. For reasons of simplicity and costliness, the most common methods of determination are radioimmunoassay (RIA) and enzyme-linked immunosorbent assays (ELISA) [28]. However, no significant differences in the salivary melatonin concentrations could be observed between passive drooling and Salivettes based on high-throughput liquid chromatography in combination with mass spectrometry (LC-MS/MS) analysis [29]. Therefore, few studies have met our inclusion criteria strictly and thus present such heterogeneous research methods.

Most of the included studies have focused on changes in the circadian rhythm of melatonin in patients with brain tumours. Joustra et al. [20] investigated if indirect indices of suprachiasmatic nucleus (SCN) functioning (such as 24-h ambulatory recordings of skin and core body temperatures, blood pressure, and salivary melatonin levels) are altered in the long term after surgery for nonfunctioning pituitary macroadenomas (NFMAs). Abnormal melatonin secretion was defined as an absence of evening rise, considerable irregularity, or daytime values >3 pg/mL. Altered melatonin secretion was observed in 41.2% NFMA patients versus 11.8% of control subjects (OR 5.3, 95% CI 0.9–30.6). Additionally, proximal skin temperature was significantly decreased during the daytime, but core body temperature and distal–proximal skin temperature gradient did not differ between groups. These findings suggest disfunction of central clock machinery, possibly caused by damage of the hypothalamic SCN by the suprasellar macroadenoma or its surgical treatment.

Interestingly, Müller et al. [22] analysed the influence of obesity on melatonin secretion in patients with childhood craniopharyngioma and with hypothalamic pilocytic astrocytoma. Craniopharyngioma patients with severe obesity had higher scores on Epworth sleepiness scale (ESS), indicating increased daytime sleepiness compared with non-severely obese ones. Salivary melatonin levels at midday and evening were similar in patients with craniopharyngioma or hypothalamic astrocytoma and in controls, without significant relationships with BMI value and tumour diagnosis. However, morning and night-time salivary melatonin concentrations were significantly correlated to BMI values and tumour diagnosis. Additionally, these samples collected at night or in the morning showed a significant negative correlation between the melatonin level and the patient’s ESS score. Severely obese craniopharyngioma patients and severely obese hypothalamic tumour patients did not differ according to the patterns of melatonin secretion. The authors speculate that hypothalamic tumours might be responsible for both daytime sleepiness and obesity.

Pickering et al. [24] conducted a cross-sectional study to evaluate the influence of craniopharyngioma or consequent surgery on melatonin secretion, and the association with fatigue, sleepiness, sleep pattern, and sleep quality. Participants did not differ in time of sleep onset; in the morning, patients woke up about an hour earlier, although the difference in time of sleep offset was only at a borderline significance. Patients suffered from increased mental fatigue and daytime sleepiness (ESS), as well as impaired sleep quality (PSQI), increased sleep latency, and daytime dysfunction. In patients, low midnight melatonin levels were related to decreased total sleep time and night sleep time, and reduced sleep efficiency and physical activity. Performed regression analysis confirmed that midnight melatonin remained independently associated with decreased total sleep time after adjustment for midnight cortisol. Additionally, low midnight melatonin in patients seemed to be correlated to increased daytime sleepiness (ESS), impaired sleep quality (PSQI), and low physical health (SF36). The authors state that craniopharyngioma patients are characterised by changes in circadian pattern, which may be associated with the altered functioning of the hypothalamic circadian and sleep regulatory nuclei.

In the other study, Pickering et al. [25] examined the circadian rhythm, fatigue, and quality of life in children with brain and cervical medulla tumours. They were grouped by tumour location involving the circadian regulatory system, defined as diencephalon, pineal gland, brain stem and cervical medulla, or other areas. Children with the first group of tumours demonstrated a lower level of nocturnal melatonin secretion related to lower inter-daily stability, higher level of fatigue, and decreased quality of life.

In contrast, in a prospective pilot study, Panciroli et al. [23] assessed if the radiotherapy dose decreased the melatonin levels as well as the quality of life and sleep in brain tumour patients. No statistically significant differences in salivary melatonin and cortisol concentrations were observed at each timepoint, as well as according to the radiotherapy dose delivered throughout the study. Additionally, the findings did not present a significant relationship between receiving radiotherapy and the quality of life and sleep.

Two studies involved changes of salivary melatonin in males with prostate cancer. In a cross-sectional study, Lozano-Lorca et al. [21] analysed the association between salivary melatonin rhythm and prostate cancer (PC) in the participants who met the selection criteria of CAPLIFE study. Both PC patients and healthy subjects had a mostly morning chronotype, as well as good sleep quality and duration. For all time points, the melatonin levels were lower in PC cases compared to the controls. The maximum peak of melatonin in both groups was observed 2 h after a bedtime. The authors conclude that PC patients could have lower melatonin concentrations than healthy men without PC, regardless of urinary symptoms or aggressiveness and extension of the tumour. In a case-control study, Farahani et al. [19] tried to find suitable biomarkers for the diagnosis of prostate cancer in serum and saliva, as well as to evaluate the diagnostic efficacy of saliva in patients with PC. Both saliva and serum melatonin levels were significantly lower in PC patients than in patients with benign prostatic hyperplasia (BPH). In contrast, serum and salivary levels of prostate-specific antigen (PSA), creatinine, urea, zinc, beta-2 microglobulin (B2M), and creatine kinase BB (CK-BB) were significantly higher in the first group. Overall, lower concentrations of the evaluated markers were observed in saliva than in serum. Moreover, in the analysis of the ROC curves, the selected biochemical parameters reached similar high predictive values in both serum and saliva.

Furthermore, Chang and Lin [17] conducted a case-control study to determine the relationships of cortisol and melatonin rhythms to sleep quality, anxiety, depression, and fatigue levels in patients with newly diagnosed lung cancer. These patients demonstrated lower salivary melatonin levels and poorer sleep quality, higher cortisol and depression levels, as well as flatter melatonin and cortisol slopes. However, the anxiety and fatigue levels did not differ significantly compared to the healthy subjects. The multivariate linear regression analysis showed that the fatigue level and cortisol slope significantly predicted sleep quality (reported as PSQI score). The lung cancer patients with flatter cortisol slope experienced a higher degree of fatigue and more severe sleep disturbance.

Salarić et al. [26] measured melatonin concentrations in unstimulated whole saliva and stimulated whole saliva in OSCC patients, as well as compared with serum levels in OSCC patients. Furthermore, they assessed the possible causal link between sleep quality (PSQI) and salivary melatonin changes. Melatonin levels in unstimulated and stimulated saliva were significantly elevated in the OSCC group in comparison to the controls. Additionally, melatonin concentrations were higher in unstimulated saliva for both groups. In contrast, sleep quality was significantly lower (i.e., PSQI significantly higher) in OSCC patients. The conducted ROC analysis allowed to distinguish patients with OSCC from healthy individuals based on melatonin levels in unstimulated whole saliva (AUC = 0.841, cut-off 0.835 pg/mL using Youden Index).

The latest study by de Bruijn et al. [18] investigated if the single-item chronotype question is related to dim light melatonin onset (DLMO), which is known as the “gold standard” for estimating the endogenous circadian phase, in (non-)Hodgkin lymphoma survivors. Most participants were more evening than morning ones (29.8%). DLMO increased gradually with at 7:45 (±1:11) p.m. for definite morning persons, 8:12 (±1:19) p.m. for both morning and evening persons, and 9:16 (±0:47) p.m. for definite evening persons. Based on the multiple linear regression analysis, the later chronotype was statistically significantly associated with higher DLMO values after adjustment for age and sex. According to the fatigued lymphoma survivors, the authors suggest that the proposed single-item chronotype is associated with dim light melatonin onset as a marker of the endogenous circadian phase and, therefore, it could be a useful alternative for more extensive morningness-eveningness questionnaires.

Although our systematic review is innovative, some limitations need to be identified. In particular, a small number of reports met the inclusion criteria in the review, and some of them have small samples without justification for their size. Selected studies showed heterogeneous groups of oncological diseases. Additionally, the study designs and methods of measuring saliva-melatonin levels were heterogeneous and thus had different sensitivity. The included studies were performed in various geographical areas. Therefore, it is important to note that melatonin secretion is influenced by solar irradiation and dietary habits.

## 4. Materials and Methods

### 4.1. Search Strategy and Data Extraction

A systematic review was conducted up to 13 March 2022, according to the Preferred Reporting Items for Systematic Reviews and Meta-Analyses (PRISMA) statement guidelines [30], using the databases PubMed, Scopus, and Web of Science. The search formulas included:

- For PubMed: (melatonin AND saliva) AND (cancer OR carcinoma OR neoplasm OR tumour OR tumor OR oncology);
- For Scopus: TITLE-ABS-KEY((melatonin AND saliva) AND (cancer OR carcinoma OR neoplasm OR tumour OR tumor OR oncology));
- For Web of Science: TS = ((melatonin AND saliva) AND (cancer OR carcinoma OR neoplasm OR tumour OR tumor OR oncology)).

Records were screened by the title, abstract, and full text by two independent investigators. Studies included in this review matched all the predefined criteria according to PICOS (“Population”, “Intervention”, “Comparison”, “Outcomes”, and “Study design”), as shown in Table 1. A detailed search flowchart is presented in the Results section. The study protocol was registered in the International prospective register of systematic reviews PROSPERO (CRD42022316612).

### 4.2. Quality Assessment and Critical Appraisal for the Systematic Review of Included Studies

**Figure 2:**
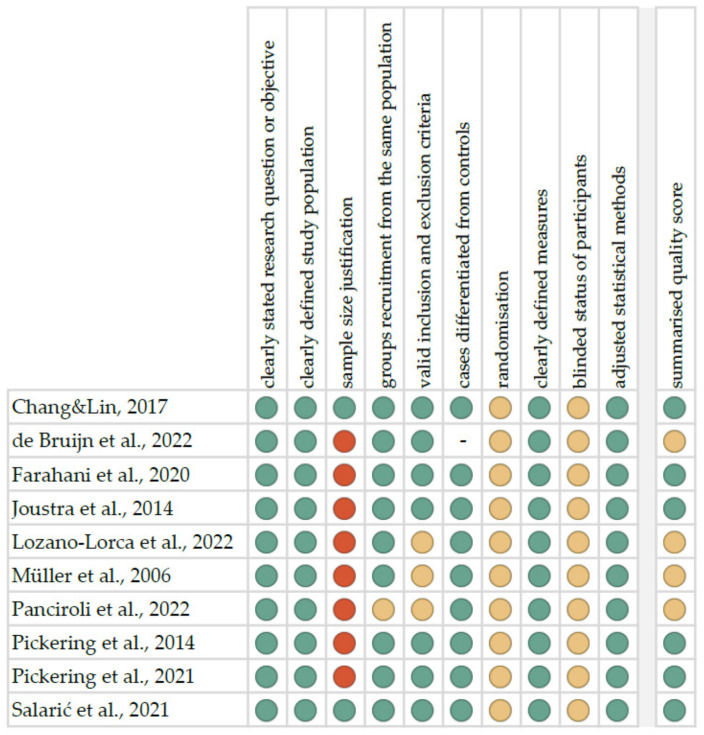
Quality assessment, including the main potential risk of bias (risk level: green—low, yellow—unspecified, red—high; quality score: green—good, yellow—intermediate, red—poor) [17,18,19,20,21,22,23,24,25,26].

The risk of bias in each individual study was assessed according to the “Study Quality Assessment Tool” issued by the National Heart, Lung, and Blood Institute within the National Institute of Health [31]. These questionnaires were answered by two independent investigators, and any disagreements were resolved by discussion between them. The summarised quality assessment for every single study is reported in Figure 2. The most frequently encountered risks of bias were the absence of data regarding sample size justification (except for two studies), randomisation (all studies), and blinding (all studies). Critical appraisal was summarised by adding up the points for each criterion of potential risk (points: 1—low, 0.5—unspecified, 0—high). Six studies (60.0%) were classified as having “good” quality (≥80% total score) and four (40.0%) as “intermediate” (≥60% total score). The level of evidence was assessed using the classification of the Oxford Centre for Evidence-Based Medicine levels for diagnosis [32]. All of the included studies have the third or fourth level of evidence (in this 5-graded scale).

## 5. Conclusions

This systematic review may suggest a link between changes in salivary melatonin levels and the occurrence of oncological diseases. However, due to the significant heterogeneity of the included reports (in terms of study design or oncological diagnosis), as well as the correlation between melatonin levels and sleep quality, it is not possible to clearly determine trends in melatonin secretion alterations in this group of patients.

## Figures and Tables

**Table 1:** Inclusion and exclusion criteria according to the PICOS.

Parameter	Inclusion Criteria	Exclusion Criteria
Population	patients with oncological diseases—aged from 0 to 99 years, both sexes	patients with other diseases
Intervention	not applicable	
Comparison	not applicable	
Outcomes	salivary melatonin concentrations or changes	only other forms of melatonin (e.g., serum)
Study design	case-control, cohort, and cross-sectional studies	literature reviews, case reports, expert opinion, letters to the editor, conference reports
	published after 2000	not published in English

**Table 2:** General characteristics of included studies.

Author, Year	Setting	Study Group (F/M) *	Control Group (F/M) *	Oncological Diagnosis	Inclusion Criteria	Exclusion Criteria	Sleep Parameters	Other Parameters
Chang&Lin, 2017 [17]	Taiwan	40 (9/31), 66.92 ± 11.01	40 (9/31), 66.68 ± 10.84	lung cancer	patients whose diagnosis was based on their first tissue biopsy; those who had not received lung-cancer-related treatment and were able to communicate	patients who were shift workers before hospitalisation because shift work can negatively impact the circadian clock, who were too weak to complete a questionnaire interview or submit a salivary sample, or who had a mental disorder	Pittsburgh Sleep Quality Index (PSQI)	Hospital Anxiety and Depression Scale (HADS), Brief Fatigue Inventory (BFI)
de Bruijn et al., 2022 [18]	the Netherlands	47 (31/16), 44.6 ± 12.8	NA	lymphoma	primary diagnosis of Hodgkin lymphoma or diffuse large B-cell lymphoma ≥2 years before study entry; moderate-to-severe fatigue since diagnosis and/or treatment; aged 18–70 years	other factors that could have affected acute fatigue or circadian rhythms	Pittsburgh Sleep Quality Index (PSQI)	visual analogue scale for fatigue (VAS-Fatigue)
Farahani et al., 2020 [19]	Iran	20 (0/20), 50.95 ± 2.76	20 (0/20), 50.45 ± 2.61; with benign prostatic hyperplasia	prostate cancer	age between 50 and 55 years; histologically confirmed diagnosis; absence of malignant neoplasms and infections; no history of oral or dental diseases	metastatic disease or already treated tumors, previous chemotherapy or radiotherapy, systemic diseases, oral or salivary-gland diseases	-	-
Joustra et al., 2014 [20]	the Netherlands	17 (8/9), 54 (26–65)	17 (6/11), 52 (30–63)	nonfunctioning pituitary macroadenoma	stable and adequate substitution of pituitary insufficiencies for at least 6 months; age of 18–65 years	patients using hypnotics or psychotropic medication; patients suffering from conditions that may alter circadian rhythmicity, i.e., sleep disorders, depression, hypertension, dyslipidemia, and diabetes mellitus	Total sleep time, Sleep efficiency; Berlin Questionnaire, Clinical symptom score, Epworth Sleepiness Scale,	Hospital Anxiety and Depression Scale, Short Form-36; skin and core body temperature, 24-h blood pressure
Lozano-Lorca et al., 2022 [21]	Spain	40 (0/40), 67.0 ± 7.3	38 (0/38), 67.5 ± 5.5	prostate cancer	newly diagnosed with histological confirmation, between 40 and 80 years old and resided in the coverage area of the reference hospitals for 6 months before recruitment in CAPLIFE study, before the first treatment for the disease	NR	Pittsburgh Sleep Quality Index (PSQI)	-
Müller et al., 2006 [22]	Germany	craniopharyngioma: BMI < 4SD: 49 (24/25), 16.1 (6.0–33.2), BMI ≥ 4SD: 30 (15/15), 16.6 (5.8–33.0); hypothalamic tumour: BMI < 4SD: 15 (5/10), 10.5 (4.8–25.6), BMI ≥ 4SD: 4 (1/3), 7.4 (6.3–8.5)	BMI < 4SD: 16 (8/8), 10.1 (4.4–24.0), BMI ≥ 4SD: 14 (3/11), 14.9 (7.4–15.5)	craniopharyngioma or hypothalamic pilocytic astrocytoma	NR	patients with missing data on ESS and melatonin secretion due to non-compliance or technical problems in collecting nocturnal saliva samples	German version of the Epworth Sleepiness Scale	BMI
Panciroli et al., 2022 [23]	Spain	12 (2/10), median = 48.5	8 (5/3), median = 38.5	brain tumors treated with radiotherapy	patients with brain tumors ≥ 18 years old before and after having received radiotherapy close to the pineal gland region	NR	Pittsburgh Sleep Quality Index (PSQI), Epworth Sleepiness Scale (ESS)	European Organization for Research and Treatment of Cancer (EORTC) quality of life questionnaires (QLQ) C30 and BN20, Distress Thermometer, Mini-Mental State Examination (MMSE)
Pickering et al., 2014 [24]	Denmark	15 (6/9), 51.4 (18.2–70.2)	15 (6/9), 51.6 (22.6–70.0)	craniopharyngioma	patients treated for former craniopharyngiomas aged 18–70 years	insufficient substitution of pituitary hormone deficiencies within 6 months before inclusion, total blindness with complete lack of perception of light and form, clinically significant liver or renal disease, use of non-steroidal anti-inflammatory drugs, b-receptor antagonists, antidepressants that affect serotonin, active cancer, epileptic seizures, working night shift, breast feeding, pregnancy and alcohol, travelling across time zones or drug abuse	Pittsburgh Sleep Quality Index (PSQI), Epworth Sleepiness Scale (ESS)	Multidimensional Fatigue Inventory, Medical Outcomes Study 36-Item Short-Form Health Survey
Pickering et al., 2021 [25]	Denmark	48 (19/29), 12.2 (7.6–16.2)	20 (7/13), 11.9 (8.0–16.3); with other brain tumours	brain tumours categorised by location involving the circadian regulatory system, defined as involving the diencephalon, pineal gland, brain stem, posterior fossa tumours compressing the brain stem or cervical medulla, or involving other locations	patients aged 0–18 years at the time of inclusion and with a previous diagnosis of a tumour of the brain or cervical medulla	tumour diagnosis, surgical tumour intervention or irradiation within six months of enrolment, and insufficient substitution of pituitary hormone deficiencies within three months of enrolment	sleep latency, duration of night sleep, total duration of day and night sleep, and sleep efficiency	Pediatric Quality of Life Inventory Multidimensional Fatigue Scale, Pediatric Quality of Life Inventory Generic Core Scales
Salarić et al., 2021 [26]	Croatia	34 (9/25), 60.6 ± 11.1	33 (10/23), 63.0 ± 13.3	oral squamous cell carcinoma (T1N0M0, *n* = 14, T2N0M0, *n* = 20)	patients with histologically verified T1N0M0 and T2N0M0 OSCC; no history of radiation therapy of the head and neck; absence of salivary, jaw and oral mucosal tissue diseases and conditions	OSCC located on the tongue root and epiglottis	Pittsburgh Sleep Quality Index (PSQI)	-

Legend: F, females; M, males; NR, not reported; NA, not applicable; BMI, body mass index; * age was presented as a mean ± standard deviation or median (1st quartile−3rd quartile).

**Table 3:** Detailed characteristics of included studies considering methods of collection and analysis of saliva.

Type of Tumour	Study	Type of Saliva	Method of Collection	Centrifugation and Storing	Method of Analysis	Other Markers
lung cancer	[17]	unstimulated whole saliva	collected through expectoration into a sterile container over a 10-min period, three times daily (9 a.m., 2 p.m. and 9 p.m.), and at least 3 mL each time	immediately stored at −20 °C	radioimmunoassay (RIA)	salivary cortisol
lymphoma	[18]	unstimulated whole saliva	collected 5 h before usual bedtime followed by one sample every sequential hour	stored at −80 °C until the analysis	liquid chromatography tandem mass spectrometry	-
prostate cancer	[19]	unstimulated whole saliva	collected for 5 min	centrifuged at 3000 rpm for 10 min and stored at −70 °C until the analysis	sandwich enzyme-linked immunosorbent assay (ELISA)	serum and salivary prostate-specific antigen (PSA), beta-2 microglobulin, creatine kinase BB, creatinine, urea and zinc; serum melatonin
prostate cancer	[21]	unstimulated whole saliva	using Salivettes on 6 timepoints: (1) 4 h before bedtime, (2) 2 h before bedtime, (3) at bedtime, (4) 2 h after bedtime, (5) 4 h after bedtime, and (6) when getting up in the morning	centrifuged and stored at −80 °C until the analysis	ultra-performance liquid chromatography-electrospray ionisation-mass spectrometry	-
brain tumour	[20]	unstimulated whole saliva	using Salivettes on two subsequent evenings (at 3, 2, and 1 h before and at habitual bedtime, and upon waking up spontaneously at night) and on the day in between (at awakening, 1 and 2 h after awakening, noon, and 3 p.m.)	kept in the dark at 4 °C until the end of assessment and stored at −20 °C until analysis; centrifuged at 1800× *g* for 15 min	radioimmunoassay (RIA)	-
brain tumour	[22]	unstimulated whole saliva	collected at different time points (morning: 6–8 h; midday: 11–14 h; evening: 18–21 h; and night time: 23–3 h) using special tubes	centrifuged and stored at −20 °C until the analysis	radioimmunoassay (RIA)	salivary cortisol
brain tumour	[24]	unstimulated whole saliva	during the 2 weeks of actigraphy, eight samples with a volume of 3 mL each collected over a 24-h period (at 12 p.m., 4 p.m., 8 p.m., 10 p.m., 12 a.m., 4 a.m., 8 a.m. and 12 p.m.)	during collection kept at 3–5 °C, then centrifuged and stored at −22 °C until the analysis	radioimmunoassay (RIA)	salivary cortisol
brain tumour	[23]	unstimulated whole saliva	collected at midnight and at 6 a.m. in the next morning, using Salivettes	NR	LC/MS/MS (liquid chromatography mass spectrometry) using ISD-MS (isotope dilute mass spectrometry)	salivary cortisol and cortisone; urinary metabolite sulfatoxi-melatonine (STM), cortisol and cortisone
brain tumour	[25]	unstimulated whole saliva	collected with Salivettes at 12 p.m., 4 p.m., 8 p.m., 10 p.m., 12 a.m., 4 a.m., 8 a.m. and 12 p.m.	during collection kept at 3–5 °C, then centrifuged at 2100× *g* for 5 min and stored at −20 °C until the analysis	enzyme-linked immunosorbent assay (ELISA)	salivary cortisol
oral cancer	[26]	unstimulated whole saliva	collected before any surgical procedure between 7 and 9 a.m. in a dark room (<3 lx) by using the specially designed saliva collecting apparatus	stored at −80 °C until the analysis	enzyme-linked immunosorbent assay (ELISA)	serum melatonin

Legend: NR, not reported; -, not applicable.

**Table 4:** The most important findings about salivary melatonin from included studies.

Type of Tumour	Study	Salivary Melatonin Findings
lung cancer	[17]	The patient group had a lower salivary melatonin level and flatter slope (*p*-value < 0.001 and < 0.001), higher salivary cortisol level and steeper slope (*p*-value < 0.001 and < 0.001), higher sleep disturbance level (*p*-value = 0.004), and higher depression level (*p*-value = 0.001). The multivariate linear regression analysis indicated that the cortisol slope (*p*-value = 0.005) and fatigue score (*p*-value = 0.032) predicted the sleep quality score (*p*-value = 0.011).
lymphoma	[18]	The mean (SD) dim light melatonin onset was at 8:42 (1:19) p.m. and the most common chronotype was more evening than morning person (29.2%). A gradual increase in dim light melatonin onset with later chronotype (i.e., evening preference) was observed, with a mean ranging from 7:45 p.m. in definite morning persons to 9:16 p.m. in definite evening persons.
prostate cancer	[19]	Serum and salivary concentrations of melatonin were significantly lower in patients with PC, compared with BPH group (*p*-value < 0.05). In both groups, salivary concentrations were lower (*p*-value < 0.05), compared with those values in serum. It was observed positive correlation between serum and salivary concentrations (*p*-value < 0.05).
prostate cancer	[21]	Melatonin levels were always lower in PC cases than in controls. On average, melatonin levels in cases were −64.0% (95% CI −73.4, −51.4) than controls. PC cases had lower amplitude, 26.0 pg/mL (SD 27.8) vs 46.3 pg/mL (SD 28.2; *p*-value < 0.001). A high amplitude was associated with a decreased risk of PC, aOR = 0.31 (95% CI 0.11, 0.86), while a late acrophase could be increased risk of PC, aOR = 2.36 (95% CI 0.88, 6.27).
brain tumour	[20]	Out of 17 NFMA patients, 7 (41%) showed at least one of the three possible abnormal parameters during daytime, i.e., either an increased distal–proximal gradient (*n* = 4), increased core body temperature (*n* = 2), or increased melatonin values (*n* = 4). Lower daytime proximal skin temperatures in NFMA patients were associated with increased daytime melatonin values (r = −0.527, *p*-value = 0.036).
brain tumour	[22]	Morning salivary melatonin levels were related to BMI (*p*-value = 0.004) and tumor diagnosis (*p*-value = 0.032). Also for nighttime salivary melatonin levels, significant relations with BMI (*p*-value < 0.001) and tumor diagnosis (*p*-value = 0.025) were detectable. Melatonin concentrations in saliva of craniopharyngioma patients collected at nighttime or in the morning showed a negative correlation (Spearman’s rho: −0.42; *p*-value = 0.001; Spearman’s rho: −0.31; *p*-value = 0.020) with the patient’s ESS score. Severely obese craniopharyngioma patients and severely obese hypothalamic tumor patients had similar patterns of melatonin secretion. Differences in terms of diurnal salivary cortisol concentrations were not detectable when patient groups and controls were compared.
brain tumour	[24]	Low midnight melatonin was associated with reduced sleep time and efficiency (*p*-value < 0.03) and a tendency for increased sleepiness, impaired sleep quality, and physical health. Midnight melatonin remained independently related to sleep time after adjustment for cortisol. Three different patterns of melatonin profiles were observed: normal (*n* = 6), absent midnight peak (*n* = 6), and phase-shifted peak (*n* = 2). Only patients with absent midnight peak had impaired sleep quality, increased daytime sleepiness, and general and mental fatigue.
brain tumour	[23]	No statistically significant differences in morning and evening melatonin levels were found according to the radiotherapy dose delivered throughout the study.
brain tumour	[25]	Children with tumours involving the circadian regulatory system typically had a lower melatonin peak (*p*-value = 0.06) and experienced significantly more fatigue and poorer quality of life. Low melatonin profiles were observed in 31% and 4% had a phase-shifted daytime peak, compared with 14% and 0%, respectively, in children with tumours located elsewhere. Children with low melatonin profiles had significantly lower inter-daily stability than those with normal profiles.
oral cancer	[26]	Melatonin levels in both unstimulated and stimulated whole saliva were significantly higher in the OSCC group. Sleep quality was significantly lower in patients with OSCC (*p*-value = 0.0001). ROC analysis was found to be significant (*p*-value < 0.001) in evaluating melatonin concentration limit in diagnosing OSCC. The expected relationship between sleep quality and salivary melatonin levels in OSCC patients was not observed.

Legend: SD, standard deviation; PC, prostate cancer; BPH, benign prostatic hyperplasia; NFMA, nonfunctioning pituitary macroadenomas; CI, confidence interval; aOR, adjusted odds ratio; BMI, body mass index; ESS, Epworth sleepiness scale; OSCC, oral squamous cell carcinoma; ROC, receiver operating characteristic.

## Data Availability

Data are available on request from the corresponding author.
